# Lessons for improved COVID-19 surveillance from the scale-up of malaria testing strategies

**DOI:** 10.1186/s12936-022-04240-4

**Published:** 2022-07-20

**Authors:** Genevieve Kerr, Leanne J. Robinson, Tanya L. Russell, Joanne Macdonald

**Affiliations:** 1 Genecology Research Centre, School of Science and Engineering, University of the Sunshine Coast, Sippy Downs, QLD 4556 Australia; 2 Burnet Institute, Melbourne, VIC Australia; 3 Australian Institute of Tropical Health and Medicine, James Cook University, Cairns, Australia; 4 CSIRO Synthetic Biology Future Science Platform, GPO Box 1700, Canberra, ACT Australia

**Keywords:** Malaria, COVID-19, *Plasmodium*, Rapid diagnostic test, RDT, Asia Pacific, LMIC, GMS

## Abstract

Effective control of infectious diseases is facilitated by informed decisions that require accurate and timely diagnosis of disease. For malaria, improved access to malaria diagnostics has revolutionized malaria control and elimination programmes. However, for COVID-19, diagnosis currently remains largely centralized and puts many low- and middle-income countries (LMICs) at a disadvantage. Malaria and COVID-19 are infectious diseases that share overlapping symptoms. While the strategic responses to disease control for malaria and COVID-19 are dependent on the disease ecologies of each disease, the fundamental need for accurate and timely testing remains paramount to inform accurate responses. This review highlights how the roll-out of rapid diagnostic tests has been fundamental in the fight against malaria, primarily within the Asia Pacific and along the Greater Mekong Subregion. By learning from the successful elements of malaria control programmes, it is clear that improving access to point-of-care testing strategies for COVID-19 will provide a suitable framework for COVID-19 diagnosis in not only the Asia Pacific, but all malarious countries. In malaria-endemic countries, an integrated approach to point-of-care testing for COVID-19 and malaria would provide bi-directional benefits for COVID-19 and malaria control, particularly due to their paralleled likeness of symptoms, infection control strategies and at-risk individuals. This is especially important, as previous disease pandemics have disrupted malaria control infrastructure, resulting in malaria re-emergence and halting elimination progress. Understanding and combining strategies may help to both limit disruptions to malaria control and support COVID-19 control.

## Background

Over the past decade substantial progress was made to reduce the global incidence of human malarias by 37% [1], with 12 new countries being certified as malaria free since 2007 [2, 3]. Countries committed to malaria elimination span the Americas, African [4], and the Asia Pacific regions [5], with a number of countries designating a timeline to achieve elimination by 2030. However, global progress has stagnated since 2014, and the current COVID-19 pandemic puts hard-earned gains at risk in many malaria-endemic countries [6–9]. The concern is that already fragile health and economic systems could collapse under the pressures of COVID-19 lockdown and control periods [10, 11], and that malaria control efforts will be delayed, resulting in a resurgence of malaria in endemic regions [12, 13]. In 2020, 58% (of 64 countries) have reported at least some disruption in their malaria programmes service delivery [14].

When malaria control services are delayed or disrupted, malaria transmission has the potential to rebound very quickly. Between 1930 and 2000 malaria resurged numerous times across the globe, and 91% of these events occurred during delays in delivery of malaria control strategies [15]. For example, the Ebola virus public health emergency in West Africa 2014 resulted in malaria re-emergence in several West African countries due to health access limitations and capacity issues [6, 7, 16, 17]. The outbreak resulted in 28,652 Ebola cases [18] across 3 malaria endemic countries. In Guinea alone, the Ebola outbreak was estimated to lead to a 45% increase in the number of untreated malaria cases [19], and subsequent investigations identified 74,000 fewer malaria cases were diagnosed at health facilities [20].

Since the start of the COVID-19 pandemic there has been over 328 million COVID-19 cases, including transmission in all 87 malaria endemic countries [21]. Recognizing the threat, the World Health Organization (WHO) has provided guidance to support continuation of malaria control services throughout the pandemic to ensure gains are maintained [22]. However, the operational reality of maintaining multiple infectious disease control programmes whilst simultaneously implementing new COVID-19 diagnostics and vaccination programmes, presents a challenge for the limited infrastructure and over-burdened health workforce in many low and middle income countries (LMIC) [23, 24].

The scope of this review is to outline how malaria diagnostics have been critical to malaria control and elimination progress. The review also highlights lessons that can be learnt from malaria control programmes, particularly focusing on the fundamental need for accurate and timely testing which remains paramount to inform accurate responses COVID-19 diagnostics in LMICs. Throughout this review, examples are drawn from the Asia Pacific, specifically the Greater Mekong Subregion, to exemplify the importance of diagnostics, because this is the locality where *Plasmodium falciparum* resistance is emerging [25], *Plasmodium vivax* concurrently presents a major obstacle [26], and a high proportion of these countries were targeting malaria elimination in the region by 2030 [27].

## Critical role of testing for both malaria and COVID-19 control

While the strategic responses to disease outbreaks for malaria [28] and COVID-19 [29] are dependent on the ecologies of each disease, the fundamental need for accurate and timely testing remains paramount. Accurate and timely diagnosis is fundamental to building strong surveillance systems that collect and analyse disease-specific data to facilitate informed public health practice [30], and highly specific and sensitive diagnostic tests are required to achieve this. There are a range of in vitro diagnostic tests available. These can be laboratory-based, such as polymerase chain reaction (PCR) testing, where samples are sent to a central laboratory for analysis. Alternatively, tests can be performed near, or at, the point of patient care, such as rapid diagnostic/antigen tests (RDTs/RATs). For any particular disease, the type of diagnostic test implemented in a country can be largely driven by the countries’ economic status. This has often led LMICs to rely on presumptive diagnosis due to cost, accessibility and/or diagnostic quality of available diagnostic services [31, 32]. Reliance on presumptive diagnostics can hinder disease control efforts [33–36] and, therefore, testing before treatment is critical for the eradication of diseases.

## Diagnostics used to improve malaria control and elimination

The most accurate diagnostic technology for malaria is laboratory-based PCR testing, however, this tool is rarely used LMIC malaria control programmes [40–42]. Lack of reliance on PCR testing is mainly due to delays in shipping samples to centralized testing facilities, which has knock-on effects to the timeliness of reporting of results. However, PCR testing is also difficult to implement due to the lack of available PC2 laboratories, long testing turnaround times, high cost, and expensive equipment requirements [40–42]. Therefore, malaria PCR is often reserved for sentinel surveillance or research settings [40–42]. Instead, the most commonly used LMIC laboratory-based diagnostic for malaria is microscopy, which is recognized as the gold standard for malaria field diagnostics [27, 49]. However, maintaining technicians skilled in malaria microscopy is increasingly difficult in health clinics [50] and limits the utility of this tool.

Due to the difficulties of performing PCR and microscopy, malaria control programmes have instead evolved to rely more heavily on point-of-care malaria RDTs, which provide simplicity and ease of use, fast results, portable testing capability, and the ability to perform testing with very little operator training requirements [51]. Compared to quality assured microscopy and PCR, RDTs can be less sensitive and specific [52], although the gains made by having a point-of-care test that is economical and can provide rapid results, are considered to outweigh any loss in diagnostic accuracy. The RDTs can be delivered and used by Community Health Workers (CHWs) in villages and communities, preventing the need for patients to come to a pre-established clinic and thereby reducing the period in which patients are infectious and transmitting disease [53, 54]. Additionally, in these remote regions, RDTs replace what was a sole dependency on presumptive clinical diagnosis [53, 55], which has the potential to reduce over-diagnosis and indiscriminate use of anti-malarial drugs for managing all febrile patients [33–36]. Indeed, RDTs are recommended over microscopy in occupationally exposed groups and during outbreaks [53, 54, 56], thus making RDTs highly favourable to coincide with malaria elimination strategies.

During in the 1990s, the introduction of RDTs provided the first malaria field diagnostic solution apart from microscopy. However, first-generation RDTs were a novel technology undergoing development and effectiveness trials with product quality varying significantly amongst the 60 manufacturers [57]. In particular, there was no quality assurance programmes for RDTs, resulting in poor performance (sensitivity, specificity, heat durability) and variations between products and lots [49, 58]. Unfortunately, suspicion surrounding the new technology grew, with communities and CHWs having little confidence in using RDTs [31, 32]. To mitigate these suspicions, a process to provide quality control transparency to the public was implemented. The “Malaria Rapid Diagnostic Test Performance” report was created and published by the WHO for public viewing. Each round of product testing would present laboratory-based data analysis for each RDT and identify the ones that met quality control criteria [59]. From these reports, informative decisions could be made on RDT selection, improving malaria diagnostics globally. However, while these quality control programmes were eventually implemented [34, 52, 59, 60], many CHWs and other health workers still rely on presumptive diagnosis due to their lack of confidence in RDTs. Additionally, health workers may avoid using RDTs or ignore negative results due to conflicts with their knowledge, patient pressure, risk of putting high risk groups in potential danger, RDT stockouts, to save unnecessary costs or limited staffing capacity [34, 60]. Notably, this impacts not only the use and acceptance of RDTs, but also follow up treatment relating to negative RDTs. In these situations, negative RDTs may incorrectly be treated as positive by a CHW if symptoms suggest the latter, resulting in the potential of other diseases resurging and patients becoming more ill due to incorrect treatment [36]. The lack of quality control frameworks during the early stage of RDT development is highly similar to the beginnings of COVID-19 RAT test development. The WHO has since released COVID-19 RAT kit assessment guidance [61] to assist countries in assessing which RDTs would meet their requirements. These experiences highlight that while it is imperative to continue research into newer and better testing methods, hasty application of poor tests could also hinder control efforts.

For malaria, efforts to re-educate CHWs and provide quality training and understanding, in addition to the RDT evaluation programmes, have greatly improved compliance to RDT procedures [36, 62, 63]. As such, there is a need for ongoing country-led capacity building and training that support the use of the RDTs, similar to that used to eliminate malaria in Sri Lanka in 2016 [64] and China in 2021 [65]. These improvements, in combination with improved RDT sensitivity and specificity, could help RDTs provide further significant progress in reducing the global malaria burden [35, 66]. However, the delay in RDT confidence and implementation in LMICs has highlighted the importance of appropriate quality control and assessment processes. It is critical that similar COVID-19 RAT roll-outs are held to the same quality processes, to prevent undermining of confidence in RDT usage in LMICs.

## The role of testing to mitigate risks to malaria control

While all malaria endemic regions have their own unique complications to malaria control, looking specifically at the Asia Pacific provides an opportunity to analyse several complications and consider whether the cautions, strategies and successes are applicable to other malaria endemic regions.

## Critical role of testing to halting the spread of artemisinin resistance

Anti-malarial drug resistance is an emerging challenge within the Asia Pacific region. Resistance to artemisinin-based combination therapy (ACT) has been detected in *P. falciparum* species in five countries within the region. Most ACT resistance is found in the Greater Mekong Subregion (GMS) with up to 4 of the potential 6 artemisinin-based combinations failing, predominantly along its international borders and near forested areas [67], with those most affected being migrant or seasonal workers [68]. If it were to spread to highly endemic regions such as Africa the effects would be catastrophic [69].

Issues that have contributed to the emergence of resistant parasite strains include over diagnoses, continual use of substandard or counterfeit ACT [70], slow-clearing ACT [71], poor adherence to ACT treatment programme, inadequate follow-up of cases, and large scale use of artemisinin-based monotherapy, particularly in China [72, 73]. However, strategies to rapidly detect and eliminate these resistant strains using point of care RDTs to screen and target endemic hotspots have been effective [74], primarily because the RDTs did not require a centralized facility to conduct testing. Thus, cases and hotspots could be swiftly traced, treated and controlled. It is critical that the remaining effective artemisinin-based combinations are reserved for treating confirmed cases only, so as to not encourage new ACT resistant strains. This aligns with the region’s current malaria elimination strategy and creates less strain on international relationships [68].

## Testing strategies contributing to malaria elimination

In 2015, the WHO Global technical strategy for malaria 2016–2030 recommended that all cases of suspected malaria should have a parasitological test (microscopy or RDT) to confirm the diagnosis, with a move away from presumptive treatment. This recommendation could only be facilitated by the availability of RDTs. The strategy transforms malaria surveillance into a core intervention, irrespective of the stage of malaria elimination, which is essential for tracking cases and responding to data received. All individuals with suspected malaria are tested, which is expected to enhance the quality and timeliness of case reporting. As transmission is reduced the information required becomes more and more granular, which includes documenting elimination through continuous surveillance and reporting.

As malaria transmission reduces it becomes more heterogenous and there is a need to stratify responses. A highly effective strategy that can be used across bordering nations to enhance malaria elimination in these settings is to focus investigations, including active case detection around an index malaria case known as reactive case detection (RACD) [75–77]. RACD testing is possible by utilising high-quality RDTs for immediate results [78]. This allows for all contacts of an infected individual to be swiftly and accurately tested, reducing the likelihood of further transmission. Other strategies that are currently being used for malaria elimination include passive case detection (PCD) and active case detection (ACD). PCD is achieved by identifying cases in symptomatic individuals who present at a health facility. ACD, on the other hand, tests a high-risk population group, rather than only people who present at a health facility, in the attempt to identify asymptomatic infections and screen hard to reach people groups [77]. What is important to highlight is that both PCD and ACD strategies used in elimination programmes benefit strongly from the use of RDTs, and their success has been attributed to quality and prompt testing.

Unfortunately, even with advancements in diagnostic technology and strategy developments, presumptive treatment is still commonly practiced in endemic LMICs [79]. Decreasing reliance on presumptive treatment is necessary for reaching elimination goals. This is achieved through a strong emphasis on implementing elimination strategies at a community level, along with training and monitoring, which has been suggested to improve testing compliance [77, 79, 80]. However, in the event of capacity issues where diagnostic testing may not be available, as a temporary measure, presumptive treatment should be utilized to ensure the safety of high-risk people groups (such as children younger than 5 years and pregnant women) [22, 81].

## Implementing learnings for improved testing of COVID-19 in malarious countries

As mentioned above, the development of point-of-care malaria testing through RDTs revolutionised malaria control programmes. There has been 3.1 billion RDTs sold between 2010 and 2020, with sales in this period increasing each year. In this same period, malaria control programs in sub-Saharan African have distributed 2.2 billion RDTs [82]. Prior to the roll-out of RDTs, the majority of the population at-risk of malaria, had very limited access to malaria diagnostics, often having to travel for at least a day to reach a centralized facility with diagnostic capacity [83]. The advent of RDTs, which provided decentralized and low-tech testing tools with rapid result and turn around, facilitated health care to move away from a mainly symptomatic diagnosis to rely on test results, even at the community level in remote areas [53, 55]. Improved access to malaria diagnostics at the health facility level has significantly contributed to the global reduction in malaria cases [75, 76, 84, 85]. This was supported through normative guidance provided by the WHO to promote that all suspected malaria patients should be tested with an approved diagnostic before being provided treatment [81].

## RDTs for improved detection of COVID-19 in rural and remote populations

In LMICs, the primary focus at the beginning of the COVID-19 pandemic was to scale up PCR-based COVID-19 testing. This is despite PCR testing having a 2–5 day turn-around time from test to result, which can increase to weeks in low-and-middle-income countries (LMIC) [37–39] with limited PCR testing resources. The capacity for PCR-based COVID-19 testing has heavily leveraged existing laboratory capacity. In many countries this has been limited only to major cities where laboratories are situated [44, 45, 86]. This is of concern in LMIC malaria-endemic countries where up to 80% of the population can live in rural and remote communities [23] resulting in a lack of widespread access to COVID-19 testing in these nations [46]. The massive effort to establish and/or expand PCR laboratories across the globe for COVID-19 testing, is being reported to occur at the expense of re-allocating staff from other disease management programmes to support the operations of these facilities [6, 7, 47, 48]. This is one of the major concerns for malaria endemic regions, where staff from malaria control programmes are being re-allocated to COVID-19 response activities [48, 87]. However, for LMICs in malaria endemic regions, one of the impracticalities of relying on laboratory based COVID-19 testing has been a lack of availability of skilled laboratory personnel to perform testing.

Fortunately, the move towards point of care COVID testing is now well underway with the development of both immunoassay and rapid-antigen (RAT) COVID-19 RATs [88]. Field testing of these COVID-19 RATs have correlated results with laboratory-based RT-PCR testing, reaching 99–100% specificity, 22–100% sensitivity [89–97] and their epidemiological outcomes can be comparable to that of PCR [43]. With the use of COVID-19 RATs increasing to up to 87% [98] of COVID-19 of tests, it has been proposed that a 100% COVID-19 RAT test regime should be acceptable [43]. However, as experienced during malaria RDT development, to obtain accurate testing for COVID-19 Ag RATs and avoid false-negative results, high quality specimens must be obtained [99], as well as quality-tested kits, which can be difficult to maintain in LMICs [100, 101]. Importantly, effective screening for COVID-19 has been demonstrated to largely depend on increasing the frequency of testing and reporting speed, and only marginally on high test sensitivity [38]. Large-scale testing and contract tracing has been highly effective, with some reporting an 8% decrease in COVID-19 mortality rate with the addition of one test per 100 people [102]. In addition, the global demand for testing kits has created a shortage of laboratory testing products [99], making alternative products such as RDTs an increasingly viable option. Indeed, there are now 22 RDTs/RATs that have been approved for home testing in Australia [103].

Portable and/or automated molecular testing for middle-level LMIC health hubs.

Another interesting development for improved testing at the point of care (POC) is the development of highly automated and/or portable molecular diagnostic tests, which provide the higher sensitivity of detection (95–100% compared to RT-PCR for COVID-19 [104]; 80–99% compared to RT-PCR for malaria [105]) associated with laboratory based testing. Portability can be provided either through the use of miniature portable machines for performing traditional PCR, or using new isothermal amplification detection methods [106, 107] that require simpler machines to operate than the machines required for performing PCR. However, all laboratory based molecular tests still have turnaround time issues and usually either require highly skilled personnel to perform the tests, or (if automated) come with associated higher equipment costs as well as the ongoing cost of testing cartridges [108]. These requirements preclude accessibility for rural and resource-limited areas, but have potential to provide a middle-level deployment in LMIC health centre hubs [107, 109, 110]. To date, 12 automated PCR and three isothermal molecular tests for COVID-19 are approved under emergency use authorisation by the Food and Drug Administration [111] for use at POC. Isothermal molecular testing for malaria remains reserved for research purposes only as recommended by the WHO [112].

## Point of care COVID-19 testing could benefit both COVID-19 and malaria mitigation

Rural and often poorer populations living beyond the span of routine clinical health care and monitoring systems are more susceptible to malaria infection [113–115]. Similarly, these same rural communities tend to have many migrant workers thus making these vulnerable groups susceptible to COVID-19 spreading as well [116, 117]. Initial COVID-19 containment efforts saw a huge influx of returning migration workers as national boarders were closed [117]. This not only attributed to up to 85% of COVID-19 cases in some Asia Pacific countries [118] but also had the potential to jeopardize malaria elimination gains made along the GMS [22] by large populations crossing boarders without correct diagnostic treatment of febrile illness. Prompt border control and quarantine efforts dramatically helped to curb further COVID-19 outbreaks in neighbouring countries [119], thus initially reducing the introduction of the disease. However, ongoing transmission is expected to be under-reported due to poor access to testing within the region [102, 109, 120]. Thus, improving access to COVID-19 testing in rural and poorer populations is critical for detecting and identifying transmission chains to reduce disease spread and mortality in these vulnerable populations. While the approach to using testing tools may be different between containment settings and preventing introduction, the tool used could be the same. In learning from malaria programmes, RDTs have benefited both control and elimination programmes—which have parallels to containing transmission and preventing introduction.

Improved access to malaria diagnostics has also contributed to the success of malaria control and elimination programmes in malaria endemic countries. As most infections in these elimination settings are in geographical hotspots [113], RDTs have allowed testing and infection tracing to be actively applied to suspected people groups without the need for a centralized sophisticated testing facility [75, 76, 85], using active case detection, mass screening and RACD strategies [27, 121, 122]. By learning from the successful elements of malaria control programmes, it is clear that improving access to point-of-care testing strategies for COVID-19 will provide a suitable framework for COVID-19 diagnosis in malaria-endemic countries and will strengthen the delivery of health care [123, 124]. This is not to say there will not be new challenges associated with co-implementing routine malaria and COVID-19 testing. There will need to be increased training for the different sample collection methods, interpretation of tests, treatments and responses. However, as mentioned earlier, in malaria endemic countries, staff from malaria control programmes are being reallocated to COVID-19 response activities and, therefore, are receiving this training along with associated costs of equipment and time [48, 87], irrespective of co-implementation of testing routines. This provides an opportunity to optimize control strategies by integrating and offering COVID-19 testing and tracing strategies alongside current malaria testing and tracing strategies, allowing LMICs to leverage existing trained staff for maximum efficiency.

In malaria-endemic countries, an integrated approach to point-of-care testing for COVID-19 and malaria would provide bi-directional benefits for COVID-19 and malaria control, particularly due to their paralleled likeness of symptoms, infection control strategies and at-risk individuals. Since the beginning of COVID-19 pandemic there has been a reduction of reported malaria cases in endemic countries, with as high as 99% less cases being reported compared to previous years [125, 126]. This may be due to a breakdown in reporting. However, there are several other factors that may have contributed to this reduction, such as symptomatic individuals being hesitant to visit health facilities due to fear of catching COVID-19 or stigma associated with being diagnosed with COVID-19, which suggests that COVID-19 infected individuals may not be seeking medical assessment [125, 127]. Various studies have also described that reported COVID-19 fatality rate is significantly lower in malaria endemic than non-malaria endemic regions which may be attributed to a number of factors including lower capacity for testing, a lower mean population age or possibly cross-immunity or shared immunodominant epitopes between *P. falciparum* and COVID-19 [128, 129].

Improved POC testing may present an initial increase in reported COVID-19 cases, as echoed in the introduction of RDTs for malaria cases [130]. However, identifying new cases is critical for control and reduced transmission. A diagnosed malaria case can be treated to eliminate further transmission. For COVID-19, a positive diagnosis enables isolation of infected individuals and protection of vulnerable people and can lead to behavioural changes to reduce transmission. Such testing can empower individuals to make informed choices about their behaviour, to further reduce the spread of COVID-19 [131]. Detection of the two diseases may have different focuses, elimination vs reduced transmission, yet increased POC testing remains vital for both.

Moreover, digital versions of the District Health Information Systems (DHIS2) have already been rapidly adapted by many countries to capture COVID-19 data, and provides a foundation for programme management [132]. Concurrent digitalized data can be further improved by implementation of RDT reader software applications. These apps have the potential to support both malaria and COVID-19 antigen testing by improving the quality of results interpretation [133] whilst linking test results into national surveillance systems [134].

## Conclusion

Point-of-care- testing for COVID-19, malaria and other infectious diseases, are more important now than ever before. Health facilities have reported dramatic declines in malaria infections since the COVID-19 pandemic began, suggesting changes in health-seeking behaviour for febrile illness. In malaria-endemic populations, an integrated approach to point-of-care testing for COVID-19 and malaria would provide benefits for both COVID-19 and malaria control. This strategy would not only be more operationally feasible to implement, it could also help to maintain malaria elimination goals by working with existing health and malaria programmes, rather than extracting resources from established malaria programmes to assist in COVID-19 control. Further development of highly specific and sensitive RDTs for both malaria and COVID-19, that are equivalent to microscopic detection (for malaria) and even PCR detection (for COVID-19 and malaria), could potentially provide detection improvements to reach all malaria infected populations and provide more accurate results for treatment.

## Data Availability

Not applicable.

## Abbreviations

ACD: Active case detection
ACT: Artemisinin-based combination therapy
CHW: Clinical health worker
COVID-19: Coronavirus disease 2019
DHIS2: District Health Information Systems
GMS: Greater Mekong Subregion
JCU: James Cook University
LMIC: Lower and middle income countries
PCD: Passive case detection
POC: Point of care
PCR: Polymerase chain reaction
RACD: Reactive case detection
RAT: Rapid antigen test
RDT: Rapid diagnostic test
RT-PCR: Real time polymerase chain reaction
USC: University of the Sunshine Coast
WHO: World Health Organization
